# Behavioral and Neurophysiological Signatures of Benzodiazepine-Related Driving Impairments

**DOI:** 10.3389/fpsyg.2015.01799

**Published:** 2015-11-26

**Authors:** Bradly T. Stone, Kelly A. Correa, Timothy L. Brown, Andrew L. Spurgin, Maja Stikic, Robin R. Johnson, Chris Berka

**Affiliations:** ^1^Advanced Brain Monitoring, Inc., Carlsbad, CAUSA; ^2^National Advanced Driving Simulator, Center for Computer Aided Design, The University of IowaIowa City, IA, USA; ^3^College of Pharmacy, The University of IowaIowa City, IA, USA

**Keywords:** simulation, benzodiazepines, impairment, driving, EEG, neurophysiology, cognitive assessment

## Abstract

Impaired driving due to drug use is a growing problem worldwide; estimates show that 18–23.5% of fatal accidents, and up to 34% of injury accidents may be caused by drivers under the influence of drugs (Drummer et al., 2003; Walsh et al., 2004; NHTSA, 2010). Furthermore, at any given time, up to 16% of drivers may be using drugs that can impair one’s driving abilities (NHTSA, 2009). Currently, drug recognition experts (DREs; law enforcement officers with specialized training to identify drugged driving), have the most difficult time with identifying drivers potentially impaired on central nervous system (CNS) depressants (Smith et al., 2002). The fact that the use of benzodiazepines, a type of CNS depressant, is also associated with the greatest likelihood of causing accidents (Dassanayake et al., 2011), further emphasizes the need to improve research tools in this area which can facilitate the refinement of, or additions to, current assessments of impaired driving. Our laboratories collaborated to evaluate both the behavioral and neurophysiological effects of a benzodiazepine, alprazolam, in a driving simulation (miniSim^TM^). This drive was combined with a neurocognitive assessment utilizing time synched neurophysiology (electroencephalography, ECG). While the behavioral effects of benzodiazepines are well characterized (Rapoport et al., 2009), we hypothesized that, with the addition of real-time neurophysiology and the utilization of simulation and neurocognitive assessment, we could find objective assessments of drug impairment that could improve the detection capabilities of DREs. Our analyses revealed that (1) specific driving conditions were significantly more difficult for benzodiazepine impaired drivers and (2) the neurocognitive tasks’ metrics were able to classify “impaired” vs. “unimpaired” with up to 80% accuracy based on lane position deviation and lane departures. While this work requires replication in larger studies, our results not only identified criteria that could potentially improve the identification of benzodiazepine intoxication by DREs, but also demonstrated the promise for future studies using this approach to improve upon current, real-world assessments of impaired driving.

## Introduction

Driving while impaired is illegal in most countries worldwide due to the public health and safety risks associated with this behavior. Such impairment is often illegal regardless of whether it is due to alcohol, illegal/recreational drugs, or prescription drugs. Despite its illegality, the 2013 National Survey on Drug Use and Health found that 3.8% of those aged 12 or older, an estimated 9.9 million people, self-reported driving under the influence of illicit drugs within the past year (SAMHSA, 2014). In addition to legal penalties, drugged driving comes with severe health risks as well. While the rate of fatal crashes due to drunk driving has generally been declining over the years (NHTSA, 2013), nonalcoholic-drugged driving-related fatalities have increased from 16.6% in 1999 to 28.3% in 2010 (Brady and Li, 2014). Rates of accidents, in general, have shown similar trends as well. In one study of 322 vehicular accident victims admitted to a trauma center in Maryland, urinalysis and plasma results showed that 15.8% of these victims tested positive for alcohol only, 9.9% tested positive for both alcohol and drugs, while an alarming 33.5% tested positive for drugs only (Walsh et al., 2004).

Although the risk of being involved in a fatal car accident is greatly increased by drug use (Li et al., 2013), the level of risk and prevalence is not the same across all drug types. Central nervous system (CNS) depressants, as compared to stimulants, narcotics, and cannabis, are associated with the greatest risk (Li et al., 2013). A 2007 National Highway Traffic Safety Administration study randomly selected drivers at traffic stops across the US during several time points throughout the day and found that benzodiazepines were one of the most commonly encountered drugs being used in day-time drivers (Lacey et al., 2009). These data are consistent with multiple studies and one meta-analysis showing that benzodiazepines are associated with 60–80% increased risk for vehicular accidents (Dassanayake et al., 2011). Furthermore, drivers who are at fault in an accident are 40% more likely to have been under the influence of benzodiazepines than those who are not at fault (Dassanayake et al., 2011).

Due to the public health risks associated with driving under the influence of benzodiazepines, objective assessments of the cognitive, behavioral, and physiological correlates of such impairments are needed. While alcohol related impairments are predictable, and correlated to breath alcohol content (BrAC), a recent review of the relationship between plasma concentrations of benzodiazepines and driving performance did not find a clear dose–response relationship (Verster and Roth, 2013). Therefore, detection of impairment due to other substances has relied on alternative assessments performed by Drug Recognition Experts (DREs). A DRE evaluation includes observations of many factors, including (but not limited to): physical appearance, driving behaviors, vital signs, psychomotor functioning, etc (Talpins and Hayes, 2004). These evaluations have proven to be highly successful in detecting driving impairments due to drug intoxication in general, yet, out of all drug classifications, with the exclusion of alcohol, CNS depressants are the least likely (41.7%) to be accurately recognized by DREs (Smith et al., 2002). Further research on correlates of benzodiazepine impairments is needed to validate the existing criteria and provide additional signs for DREs to rely on to increase their accuracy in drug specificity. Alternative research methods and tools may be necessary to facilitate such investigations. In particular, the utilization of a cognitive and physiological assessment with time-synced neurocognitive tasks and electroencephalography (EEG) may prove to be a successful approach toward meeting this goal.

Research has shown promise for the utilization of cognitive and behavioral performance for such an assessment. In regards to cognitive task performance, benzodiazepines decrease alertness (Verster et al., 2002; Barker et al., 2004), increase reaction times (Fernández-Guardiola et al., 1984; Suzuki et al., 1995; Münte et al., 1996; Verster et al., 2002; Barker et al., 2004; Snyder et al., 2005; Leufkens et al., 2007), impair vigilance (Kožena et al., 1995), and impair various memory functions such as verbal and working memory (Linnoila et al., 1990; Rush and Griffiths, 1997; Verster et al., 2002; Barker et al., 2004; Snyder et al., 2005; Leufkens et al., 2007). These effects are dose-dependent. For example, a 0.5 mg dose of the benzodiazepine, alprazolam, significantly slowed reaction times on simple attention tasks (Snyder et al., 2005). A 1.0 mg dose also slowed reaction times on simple attention tasks and additionally decreased accuracy on tasks assessing executive functions and learning, and increased reaction times on executive and psychomotor tasks (Snyder et al., 2005). In addition to impairing cognitive function, benzodiazepines can have adverse effects on motor control (Verster et al., 2002; Barker et al., 2004) and psychomotor functioning (Linnoila et al., 1990; Rush and Griffiths, 1997; Riba et al., 2001; Barker et al., 2004; Snyder et al., 2005). Taken together, these data suggests that this class of drug may impair various driving-related skills as driving requires the coordination of both cognitive and motor skills. In fact, studies have confirmed that benzodiazepine use is directly associated with driving impairments related to control of lateral position within the lane or completely crossing into adjacent lanes or shoulders (O’Hanlon et al., 1982; Van Laar et al., 1992, 2001; Bocca et al., 1999; Verster et al., 2002; Leufkens et al., 2007), speed control (Van Laar et al., 1992; Verster et al., 2002; Staner et al., 2005), and steering (Smiley and Moskowitz, 1986). Use of this drug has also been shown to negatively impact one’s abilities to assess the surrounding environment (Verster et al., 2002; Leufkens et al., 2007; Dassanayake et al., 2011) and to slow reaction times related to driving performance (Kuitunen, 1994; Bocca et al., 1999; Vanakoski et al., 2000; Leufkens et al., 2007; Dassanayake et al., 2011).

EEG has also been utilized to measure the neurocognitive effects of benzodiazepines and driving. Benzodiazepines can affect several EEG power spectral density bandwidths linked to attention and internal processing (Buchsbaum et al., 1985; Harmony et al., 1996; Eoh et al., 2005). Research has shown benzodiazepines to be related to increases in Delta (1–3 Hz) in the bilateral frontal–temporal and temporal–occipital regions (Staner et al., 2005). Such increases in Delta, as well as Theta (3–7 Hz), have been positively correlated with fatigue while driving (Lal and Craig, 2002; Campagne et al., 2004), and could possibly serve as an indicator of benzodiazepine-related driving impairment. Benzodiazepines also decrease Alpha (8–13 Hz) band activity in the frontal, temporal, and occipital regions (Buchsbaum et al., 1985; Bond et al., 1992; Staner et al., 2005). Suppression of Alpha activity can be indicative of task-related increases in cognitive demand (Klimesch, 1999; Eoh et al., 2005) and has even been specifically linked to driving; easier simulator courses produce higher levels of Alpha than more difficult simulator courses in healthy drivers (Eoh et al., 2005). The suppression of Alpha associated with benzodiazepine use may be indicative of cognitive compensatory mechanisms being activated, leaving fewer resources for coping with higher driving demands when needed. Since increases in cognitive demand are correlated with greater likelihoods of committing errors (Fairclough et al., 2005), it is possible that the reduction in Alpha due to benzodiazepine use may also serve as an indicator for increased risk of driving errors. EEG Beta activity (13–30 Hz) is of particular interest in relation to driving abilities; as benzodiazepine use is associated with increased activity in the parietal and central regions (i.e., motor cortex) as well as in the bilateral frontal–temporal and temporal–occipital regions (Hendler et al., 1980; Buchsbaum et al., 1985; Bond et al., 1992; Staner et al., 2005). Because increased Beta activity across the motor cortex is associated with a reduction in motor movements (Baker, 2007), this could potentially be an indicator of a reduction in driving-related psychomotor functioning.

In addition to EEG metrics, ECG metrics may also be informative in assessing impairments associated with benzodiazepine use. For example, research has shown that the administration of a benzodiazepine elevates heart rate (HR; Muzet et al., 1981; DiMicco, 1987; Ueda et al., 2013). This finding could possibly be related to, and predictive of, the impairments associated with this class of drug. HR variability (HRV) may also be predictive of such impairments as its quadratic relationship with the activation of the parasympathetic nervous system (Goldberger et al., 2001) provides detail on the autonomic nervous system’s functioning through the low frequency/high frequency ratio (Camm et al., 1996; Sztajzel, 2004). In fact, research has shown promise for using this metric to detect driving errors. For example, decreased HRV is related to drowsiness while driving (Murata and Hiramatsu, 2008), as well as increases in drowsiness related driving errors (Michail et al., 2008). This class of drugs has been shown to reduce HRV (Adinoff et al., 1992; Agelink et al., 2002), which, in addition to drowsiness, may also indicate impaired cognitive functioning. To this end, research has shown that those with lower HRV show decreased accuracy, and increased reaction times, on working memory and vigilance tasks (Hansen et al., 2003). For these reasons, ECG was also time-synced to the neurocognitive tasks. In summary, these findings provide a foundation establishing that the neurocognitive, physiological, and behavioral effects of benzodiazepines can both be measured and used to detect levels of driving related impairment.

While there has been extensive research on the cognitive, behavioral, and neurophysiological effects of benzodiazepines, research is needed on whether these impairments can be utilized to predict and differentiate between who is safe to drive and who is impaired due to benzodiazepine use. The variability inherently associated with real-world, on-the-road assessments, in addition to the risk for accidents, complicates the orchestration of safe and controlled research studies of benzodiazepine-related driving impairments. Innovations in virtual environments have made driving simulators a highly valid alternative to on-the-road assessments. Furthermore, driving simulator performance has been shown to be related to, and predictive of, on-the-road driving performance (Lee, 2003; Lew et al., 2005; De Winter et al., 2009; Bédard et al., 2010). Therefore, a driving simulator is the most effective platform for conducting safe, controlled, and reproducible studies on the effects of CNS active drugs, such as benzodiazepines, on driving performance.

The current study utilized such a platform to assess the use of neurocognitive, physiological, and behavioral indicators for predicting benzodiazepine-related impairment. Although performance on laboratory-based cognitive tasks have previously been shown to be related to Standard Deviation of Lateral Position (SDLP; Verster et al., 2002), an indicator of driving performance, these tasks, alone, have not been able to predict much of the variability in SDLP (Verster and Roth, 2012). By adding real-time neurophysiology to cognitive tasks, we hypothesized that we would be able to predict variability in simulated driving performance. Since simulated driving performance predicts real-world driving, such findings would not only demonstrate the promise for this alternative approach for future research on this topic, but could also lead to further validation of, and improvement upon, assessments for drugged driving related impairments.

## Materials and Methods

### Participants

A total of *N* = 24 participants were recruited and enrolled following a two level screening process. Participants were recruited through a database registry (*n* = 7000+) whereby emails and phone calls were sent out to a randomized set of individuals who met the pre-screening criteria. Additionally, participants were recruited through emails sent to all 30000+ students/faculty associated with the University of Iowa. Prior to any participant screening visits, an initial phone screen was completed to increase the rate of participants passing the screening visit. The phone screening involved a variety of yes or no questions to determine preliminary eligibility.

If initially eligible, participants were required to come to the National Advanced Driving Simulator facility (NADS) at the University of Iowa’s Research Park (Coralville, IA, USA), for an in-person screening visit, and to complete the informed consent protocol prior to a more rigorous screening process. Once enrolled, participants were then asked to complete the following additional screening procedures: an initial urinalysis drug screen to ensure they tested negative for all drugs, a pregnancy screen (if female), a brief physical examination of vital signs (including HR and blood pressure), and a psychiatric exam using the Columbia-Suicide Severity Rating Scale (C-SSRS). After successful completion of the physical and psychiatric screening, an in-depth survey was administered that included detailed demographics and questions about the presence and extent of any preexisting abnormalities and/or mental health issues that may put the participant at a greater risk for health complications, adverse drug reactions, or interfere with the study procedures and results. All potential enrollees were then asked to complete a brief (5–8 min) drive in the simulator to assess for the propensity of simulator sickness and to familiarize them with the operation of the simulator. Following the drive, a wellness questionnaire was administered to determine their individual risk of simulator sickness; those scoring at high risk were removed from further participation.

A total of *n* = 19 participants were selected for the analysis for this study out of the originally qualified *N* = 24. The exclusions were due to: (a) having poor EEG and/or ECG data quality (<80% good data) (*n* = 1); (b) Simulator Sickness Questionnaire indicating increased proneness to simulator sickness (*n* = 1); (c) testing positive for non-approved drug within their system on orientation visit (*n* = 2); and (d) failure to comply with study protocol (*n* = 1). The final dataset (*n* = 19) was comprised of 31.6% females with 94.7% of the participants identifying as Caucasian. This dataset had a mean age of 25.3 yr (range: 18–38 year), a mean weight of 81.19 kg (*SD*: 12.85 kg), and a mean height of 176.64 cm (*SD*: 15.85 cm).

All participants received monetary compensation for taking part in the current study. Participants were paid at the end of their last visit. Payment schedules went as follows: $25 for Orientation, $115 for first experimental session, and $135 for the second experimental session. If a participant was unable to complete the experiment, they were paid for any past visits and additional $20/h for the time they completed in their last session. All procedures were reviewed by the University of Iowa IRB, and approved prior to study implementation.

### Equipment/Materials

#### Self-report Tools

Three self-report tools were used after final screening to (1) screen out those at higher risk of simulation sickness (Simulator Sickness Questionnaire; Kennedy et al., 1993); (2) confirm compliance with sleep and caffeine intake requirements (Intake Survey; Brown et al., 2014); and (3) to assess general sleepiness prior to drug administration (Stanford Sleepiness Scale; Johnson et al., 1990). As the class of benzodiazepine drugs are associated with increased fatigue, and the effects on driving are thought to stem, in part, to the fatigue related effects, sleepiness assessment is essential to ensure that the effects of the drug are being measured, rather than some unrelated fatigue issues.

#### Psychophysiology

EEG and ECG were acquired using the B-Alert^®^ X10 wireless sensor headset (Advanced Brain Monitoring, Inc, Carlsbad, CA, USA). This system had nine referential EEG channels located according to the International 10–20 system at Fz, F3, F4, Cz, C3, C4, POz, P3, and P4 and an auxiliary channel for ECG. Linked reference electrodes were located behind each ear on the mastoid bone. ECG electrodes were placed on the right clavicle and the lower left rib. Data were sampled at 256 Hz with a high band pass at 0.1 Hz and a low band pass, fifth order filter, at 100 Hz obtained digitally with Sigma-Delta A/D converters. Data was broadcasted to an iOS-Compatible Bluetooth transmitter using a wired configuration, which then transmitted the data wirelessly via Bluetooth to a host iOS device, where acquisition software then stored the psychophysiological data. The proprietary acquisition software also included artifact decontamination algorithms for eye blink, muscle movement, and environmental/electrical interference such as spikes and saturations. After obtaining head measurements, the B-Alert^®^ X10 wireless sensor headset was placed on the subject’s head, with accompanying leads, and the impedance was tested until optimal connectivity was achieved (<40.0 kΩs) prior to each of the three data collection sessions.

#### Drive Simulator

The NADS miniSim^TM^ Research Driving Simulator is a PC-based research driving simulator with powerful scenario editing and data acquisition capabilities that is based on over a decade of research and driving simulation experience at the University of Iowa’s National Advanced Driving Simulator (NADS). The miniSim^TM^ can be configured a variety of ways, including single and multi-screen displays and desktop, ¼-cab and ½ cab configurations.

#### miniSim^TM^ Data Collection Mode

The miniSim^TM^ data acquisition system (DAQ) runs every time a scenario is ran. The DAQ collects well over 100 variables for post-processing; the Runtime Measures Evaluation functionality offers the researcher the option of obtaining measures from the simulator immediately when the drive ends. Among these variables are: Mean Lane Position, Standard Deviation of Lane Position, Lane Departure, Standard Deviation of Steering Wheel Angle, Steering Entropy, Steering Bandwidth, Lateral Acceleration, Speed, Standard Deviation of Speed, Longitudinal Acceleration, and other event specific measures. The measures are reported for the entire drive. The user has total control in defining the start and end points of each event through the scenario. For the analyses presented herein, there is a focus on assessing Standard Deviation of Lane Position and Lane Departures, as these metrics have shown promise in assessing benzodiazepine-related impairments in previous studies. The Standard Deviation of Lane Position is a combination of averaging the variability in lane position across events and road conditions, as well as the variability of speed across these events (25–70 mph), and variability and driving conditions, while Lane Departure refers to any time a wheel crossed over a lane line.

A total of three scenarios were programmed with consistent events, and comparable difficulty, that included three Road type segments: Rural, Urban, and Highway; with 6–10 events in each segment. These scenarios were used equally, counterbalanced across participants so that each received two (1 per drive). The events are shown in **Table 1**.

**Table 1:** Road type segments and events.

Road type segment	Event	Description
Rural	TurnOffRamp	Transition from off-ramp to rural road
Rural	Lighted	Straight section of lighted rural road
Rural	TransToDark	Partially lighted rural road
Rural	Dark	Mixture of curves and tangents without environmental lighting
Rural	TransToGravel	Transition from dark rural to gravel
Rural	Gravel	Rural gravel road with curves
Rural	Driveway	Gravel curve past a house and driveway
Rural	GravelExtension	Rural gravel road with curves
Rural	GravelTransToRural	Transition to paved rural road from gravel
Rural	RuralStraight	Ten minute rural tangent
Highway	OnRamp	Transition from urban to interstate via ramp
Highway	MergeOn	Transition from ramp to interstate
Highway	Interstate	Divided highway with traffic in same direction
Highway	MergingTraffic	Interchange with traffic that merges and forces driver to change lanes
Highway	InterstateCurves	Divided highway with curves
Highway	ExitRamp	Transition from interstate via ramp
Urban	Pullout	Entering driving lane from parking spot
Urban	Urban General	Urban environment with curves and tangents
Urban	Green Light	Intersection with green light
Urban	Yellow	Intersection with light that turns yellow as driver approaches
Urban	Left	Intersection with left turn across traffic
Urban	UrbanCurves	Less dense urban environment with curves
Urban	UrbanEarly	Urban environment with curves and tangents

### Doses, Administration, and Design

#### Drugs

The study was conducted according to a double blind, placebo-controlled, within subject, cross-over design. Two dosing conditions existed: (1) administration of the benzodiazepine and (2) administration of a matched placebo. In order to provide an identical dose, both conditions delivered an encapsulated, identical in size (size 0) and similar in weight and appearance (non-labeled, colored-blue and opaque), pill. The benzodiazepine [1 mg instant release (IR) alprazolam] was manufactured by Mylan^®^ and obtained through the University of Iowa Hospitals and Clinics, under the DEA license of the Principal Investigator (PI). A low dose of 1mg IR alprazolam was chosen because several studies have found that this dose significantly impairs cognitive function and driving performance (Leufkens et al., 2007; Dassanayake et al., 2011). The placebo (lactose) was supplied as lactose monohydrate NF (National Formulary), manufactured by Professional Compounding Centers of America (PCCA). The placebo and the benzodiazepine were encapsulated at the University of Iowa College of Pharmacy by blinded outer capsules, made by Gallipot Inc., to ensure that administration of the drug/placebo remained blind.

#### Neurocognitive Assessment Tasks (M-AMP)

Participants were required to complete the Mobile Alertness Memory Profile (M-AMP) as part of the study protocol at three time points (orientation and as part of each of the two experimental sessions), with synchronized psychophysiology from the B-Alert X10. Using an Apple iPad^®^ (fourth generation), the tasks that were completed included:

##### 3-Choice Active Vigilance Task (3CVT)

The three choice active vigilance task (3CVT) is a 20 min long task that requires participants to discriminate one target (70% occurrence) from two non-target (30% occurrence) geometric shapes. Each stimulus was presented for a duration of 200 ms. The inter-stimulus interval was variable and changed for each quartile of the task: 1–3 s for the 1st quartile, 1–6 s for the second and third quartiles, and 1–10 s for the last quartile. Participants were instructed to respond as quickly as possible to each stimulus by selecting the left arrow for target stimuli and the right arrow for non-target stimuli. A training period was provided prior to the beginning of the task in order to minimize practice effects.

##### Visual Psycho-Vigilance Task (VPVT) and Auditory Psycho-Vigilance Task (APVT)

The Visual Psycho-Vigilance Task (VPVT) and Auditory Psycho-Vigilance Task (APVT) were passive vigilance tasks that lasted 5 min each. The VPVT repeatedly presented a 10 cm circular target image for a duration of 200 ms. The target image was presented every 2 s in the center of the computer monitor, requiring the participant to respond to image onset by pressing the spacebar. The APVT consisted of an auditory tone that was played every 2 s, requiring the participant to respond to auditory onset by pressing the spacebar.

##### Standard Image Recognition (SIR)

The Standard Image Recognition (SIR) task was used to evaluate attention and short-term memory and takes 6 minutes to complete. The IR task included both training and testing periods. During the training period, participants were asked to memorize a series of 20 target images that were presented twice per image. To ensure the participant was attending to the target images, they were required to respond to each image by pressing the left arrow key. In the testing period, the participants were then asked to identify the target images (selecting the left arrow key for targets or right arrow key for non-target, as with the 3CVT) in a field of 100 total images (20 targets/80 non-target). This task is capable of employing several different categories of images (animals, food, sports, and travel); the animals category is always used unless the participant must restart the task, in which case images from a different category are displayed. These images were used in a counterbalanced order across participants to ensure that there were no carryover effects over time for each participants.

#### Protocol

Eligible participants completed a total of three visits: orientation and two experimental sessions that were identical in procedure, differing only in dosing condition.

##### Orientation

After completion of the screener and determination of final eligibility, participants were asked to complete an initial M-AMP with synchronized psychophysiology. The 45-min session consisted of the 3CVT (20 min), VPVT (5 min), APVT (5 min), and SIR (6 min), as well as practice and transition periods. These tasks began prior to 11:00 AM in order to ensure that diurnal variation in the EEG signal did not confound results throughout the study. If participants were unable to begin these tasks prior to 11:00 AM, a second orientation visit was scheduled. The first experimental sessions were scheduled upon completion of the M-AMP.

##### Experimental Sessions

The two experimental study visits were scheduled at a minimum of 5 days apart in order to allow for an adequate drug wash-out period between study visits. Experimental sessions lasted approximately 5–6 h and started at either 7:00 AM or 8:00 AM. Upon arrival for a study visit the subject was required to provide a urine specimen for drug screening and, if female, a pregnancy test. In the case of a positive drug screen, the subject was taken home and the visit was rescheduled for a later date, taking into account the time it takes for the drug to clear the participant’s system. After urinalysis, the capsule (drug or placebo) was administered to participants accompanied with a full glass of water. Upon swallowing the capsule, a two-hour waiting period began. During this two hour waiting period, to allow for peak activity of the study drug, participants remained seated and relaxed in the subject prep room. Subjects were allowed to listen to music, read, and/or browse the internet, however continuous monitoring ensured that they did not fall asleep. Immediately following this waiting period, the M-AMP was administered to assess neurocognitive functioning. All M-AMP sessions began by 11:00 AM to ensure limited diurnal variation in the EEG signal. Once participants completed this test battery, they were moved into the miniSim^TM^ room and began their drive.

The study drive consisted of four simulated nighttime segments. Each segment lasted approximately 10 minutes and included urban, freeway, and rural roadways that included a mixture of road geometries and speeds. It should be noted that this drive was designed to provide a cross section of driving environments to assess how performance varies as driving context varies and differs (Lee et al., 2010) substantially from traditional simulated road tests utilizing only straight roads at a constant speed. Therefore, as the SDLP values are considered, it is important to note some differences relative to other published data. Most published data uses a common driving environment including straight roads at a constant speed, but the research presented herein involves a diverse environment containing a variety of driving situations. The result of this is that many of our SDLP values are not directly comparable to other methodologies. Lastly, participants completed the Simulator Sickness Questionnaire in response to their experience during the study drive.

The second experimental session was scheduled at the end of the first session. After each visit, participants were taken home by a taxi service to ensure they were not driving under any potential influence due to the study procedures.

### Statistics

One way ANOVAs (drug condition) were conducted for the drive metrics of SDLP and Lane Departures (LnDPs), both for the overall drive and for each event of the drive (i.e., Urban Yellow Light, Rural gravel Extension); Bonferroni adjustments were made for multiple comparisons. Our goal is not to identify drug use, but rather the impairments associated with drug use. For this reason, we stratified participants/sessions into “impaired” vs. “unimpaired” based on performance on SDLP and LnDPs. From this stratification we found that *n* = 1 placebo session was impaired, and *n* = 6 drug use sessions were unimpaired. We removed the placebo confounded session from further analysis, and included the “drug-unimpaired” in the unimpaired category after an initial assessment showed no significant differences between these sessions and the placebo-unimpaired group.

We then conducted one-way ANOVAs on the M-AMP data for performance and neurophysiologic metrics: EEG bandwidths, EEG wavelets, HR, and HR Variability, based on impairment. Tukey’s range test for pairwise comparisons were used for *post hoc* tests.

In order to begin to determine the predictive value of the M-AMP on driving impairment, we regressed the M-AMP data onto the SDLP and LnDP, by M-AMP task (i.e., we regressed the 3CVT metrics separate from the VPVT, APVT, and SIR metrics). We performed several forward step-wise regressions to identify the predictive power of performance and neurophysiological metrics for each of the aforementioned tasks. During the step-wise regression, in each step, a set of *F*-tests were performed as the selection criteria to determine the explanatory power of variables and to select which variables to include in the model. The metrics identified by the regressions were then used in a cross-validated 2-Class discriminate function analysis by “impairment” status (based on SDLP and LnDP as noted) to identify the potential predictive power of the neurocognitive assessment in predicting driving impairment.

To further evaluate classification accuracy, we applied a machine learning approach (boosting) to the dataset. The boosting algorithm, AdaBoost (Freund and Schapire, 1997; Viola and Jones, 2001) combines multiple weak learners into a single strong classifier. Each weak learner is a simple decision stump that depends only on a single variable from the input training vectors. The final prediction rule is a weighted majority vote of weak learners in which the weight of each weak learner is a function of its accuracy. The error of the boosted classifier drops exponentially when the weak learners’ accuracy is slightly better than random guessing. AdaBoost maintains a set of weights over the training samples to focus the training process on samples that are misclassified. This is done by increasing the weights of the training samples that are misclassified and decreasing the weights of the training samples that are correctly classified in each boosting round.

## Results

### One Way ANOVAs

#### Drive

**FIGURE 1:**
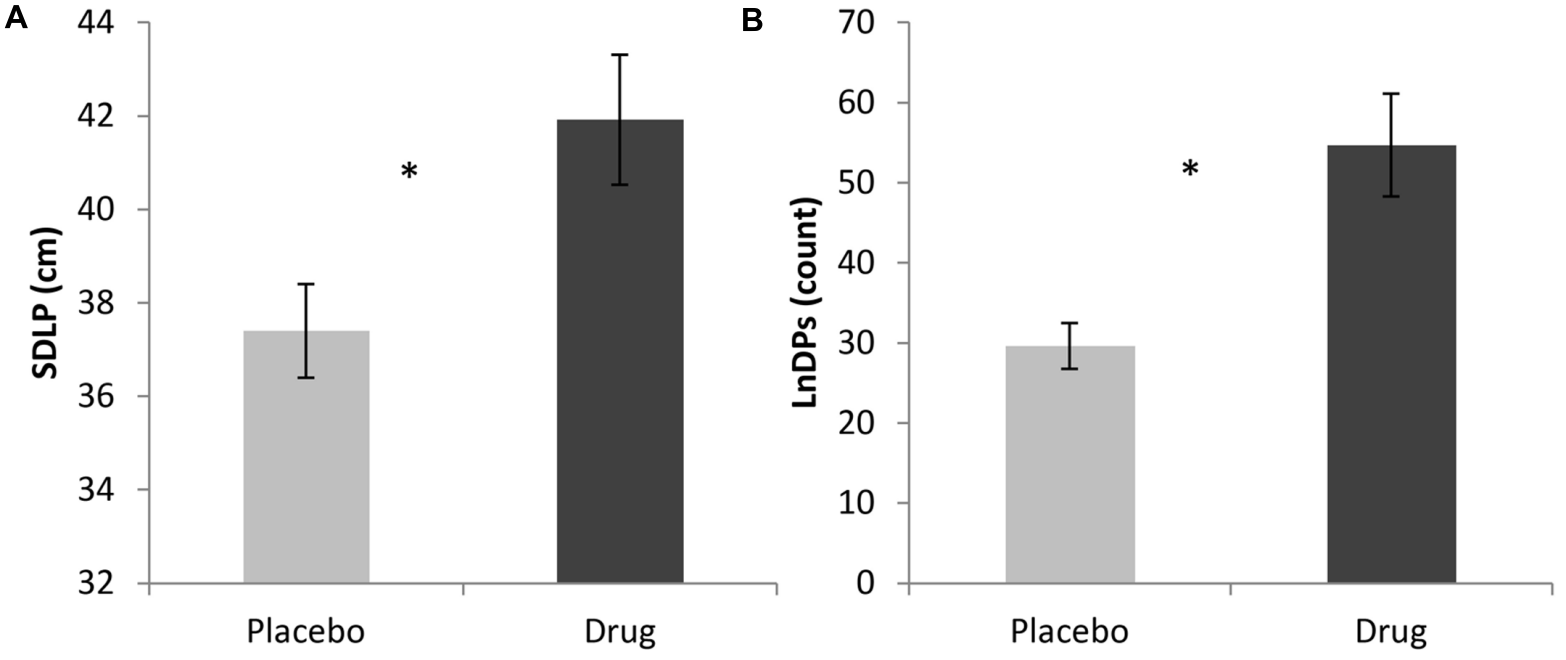
**(A)** ANOVA results of overall SDLP for Placebo versus Drug; **(B)** ANOVA results of overall LnDP for Placebo versus Drug (^∗^*p* < 0.05).

A one-way ANOVA was conducted in order to investigate the effects of the drug on SDLP throughout the driving session which revealed that those who were given the benzodiazepine (*M* = 41.76 cm, *SD* = 6.10) had significantly greater SDLP than those who were given the placebo (*M* = 37.49 cm, *SD* = 4.27), *F*(1,33) = 6.12, *p* < 0.05. For comparison, the relatively straight portion of the urban drive, when considered alone, showed the same pattern of effects with those who were given the benzodiazepine (*M* = 25.73 cm, *SD* = 9.16) having a significantly greater SDLP than those who were given the placebo (*M* = 19.19 cm, *SD* = 8.49), *F*(1,18) = 6.02, *p* < 0.05. Since the inability to maintain lane position is one of the cues officers use to predict impairment due to intoxication (NHTSA, 1997), we investigated LnDP as well. The one-way ANOVA on the sum of LnDPs for the overall drive revealed a significant difference between drug conditions, *F*(1,33) = 10.11, *p* < 0.01, with the benzodiazepine condition (*M* = 54.68, *SD* = 28.72) having significantly more LnDPs than placebo (*M* = 29.6, *SD* = 11.46). These findings are shown in **Figure 1**.

**FIGURE 2:**
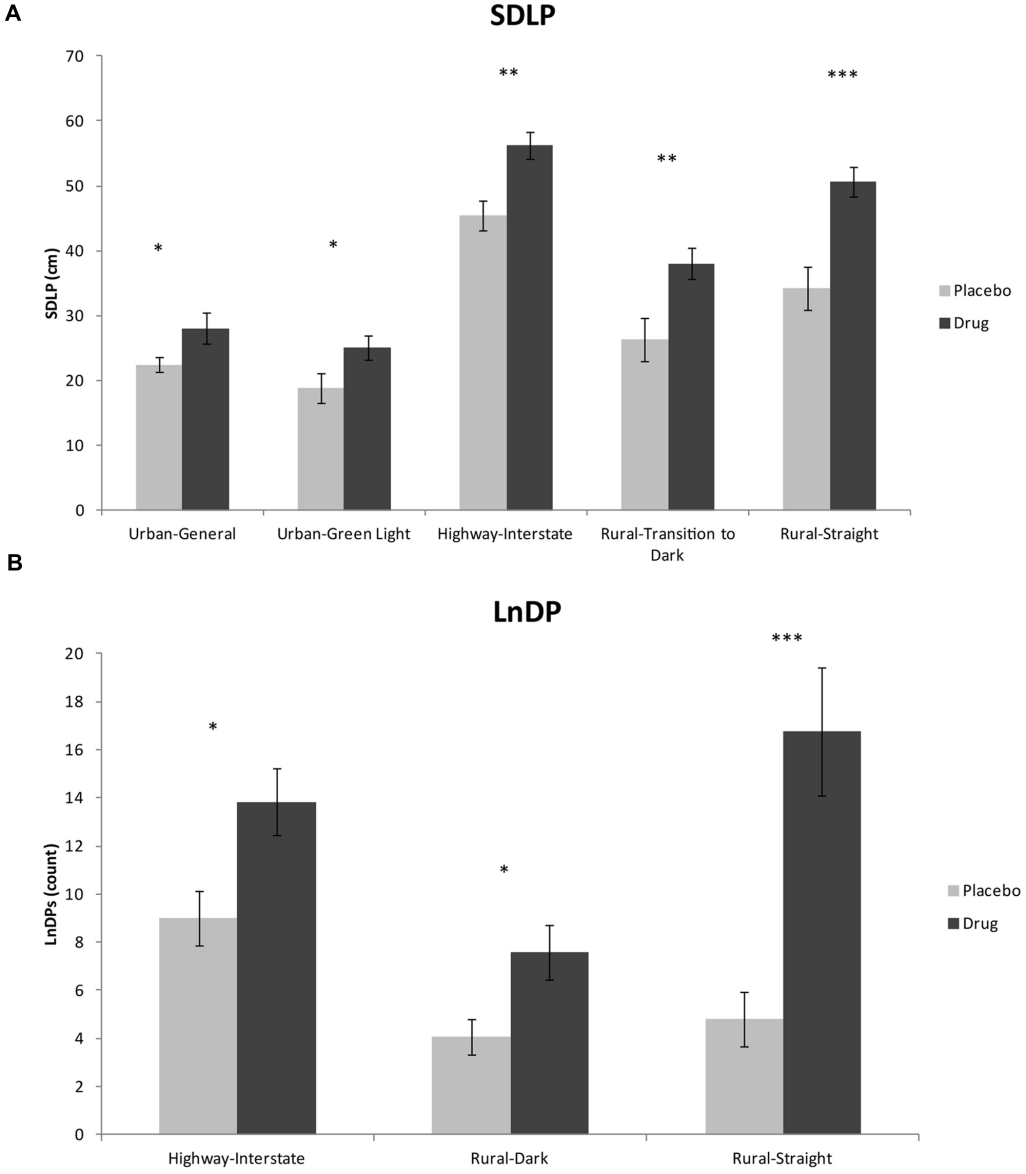
**(A)** ANOVA results of SDLP for road type-events; **(B)** ANOVA results of LnDP for road type-events (^∗^*p* < 0.05; ^∗∗^*p* < 0.01; ^∗∗∗^*p* < 0.001).

In addition to overall drive effects, ANOVAs were used to examine the events within the drive, as some are more sensitive to impaired driving than others, by design. For SDLP, we found significant differences between conditions for the following events: Urban General *F*(1,33) = 4.27, *p* < 0.05; Urban Green Light *F*(1,33) = 4.66, *p* < 0.05; Highway Interstate *F*(1,33) = 10.24, *p* < 0.01; Rural Transition to Dark, *F*(1,33) = 8.18, *p* < 0.01; and Rural Straight, *F*(1,33) = 12.81, *p* < 0.001. These data are reported in **Table 2** and shown in **Figure 2**. For LnDPs, the following events revealed significant differences between the two conditions: Highway Interstate *F*(1,33) = 6.36, *p* < 0.05; Rural Dark *F*(1,33) = 5.52, *p* < 0.05; and Rural Straight *F*(1,33) = 14.24, *p* < 0.001 (see **Table 2**; **Figure 2**).

**Table 2:** One way ANOVA – drive metrics.

Metric	Road type segment-event	Condition	Mean (cm)	Standard deviation (cm)
**SDLP**	Urban-general^∗^	Placebo	22.45	7.08
		Drug	28.03	8.34
	Urban-green light^∗^	Placebo	18.77	7.95
		Drug	25.03	8.74
	Highway-interstate^∗∗^	Placebo	45.5	6.4
		Drug	56.27	11.71
	Rural-transition to dark^∗∗^	Placebo	26.29	6.89
		Drug	37.98	14.56
	Rural-straight^∗∗∗^	Placebo	34.21	7.06
		Drug	50.65	17.15

**LnDP**	Highway-interstate^∗^	Placebo	9	4.5
		Drug	13.84	6.26
	Rural-dark^∗^	Placebo	4.07	2.94
		Drug	7.58	5.16
	Rural-straight^∗∗∗^	Placebo	4.81	4.68
		Drug	16.79	11.92

*^∗^p < 0.05; ^∗∗^p < 0.01; ^∗∗∗^p < 0.001*

#### M-AMP Impairment

One way ANOVAs were conducted to detect significant differences in performance and neurophysiology between the drug-impaired and unimpaired groups for all M-AMP tasks. The two groups significantly differed on the following physiologic metrics during the 3CVT: P3 Gamma (25–40 Hz), *F*(1,33) = 4.22, *p* < 0.05; and Midline Alpha Slow (Fz, Cz, Pz, and POz from 8 to 10 Hz), *F*(1,33) = 4.22, *p* < 0.05. For performance, we found reaction time and reaction variability to be significantly different, including: overall correct response variability, *F*(1,33) = 5.07, *p* < 0.05; variability in correct response to interference stimuli during the first quartile, *F*(1,33) = 4.45, *p* < 0.05; reaction times for correct responses across all stimuli types during the fourth quartile *F*(1,32) = 4.59, *p* < 0.05; and variability in correct response reaction times across all stimuli types during the 4th quartile, *F*(1,32) 4.67, *p* < 0.05. The mean and SD for these findings are shown in **Table 3**.

**Table 3:** One way ANOVA – 3CVT data.

Metric	Impairment group	Mean	Standard deviation
P3 Gamma (25–40 Hz)	Unimpaired	2.44	0.40
	Drug-impaired	2.20	0.19
Midline Alpha Slow (8–10 Hz)	Unimpaired	2.95	0.26
	Drug-impaired	3.19	0.41
Overall standard deviation of reaction time for correct-targets	Unimpaired	0.15	0.06
	Drug-impaired	0.20	0.06
Standard deviation of reaction times to interference stimuli (Q1)	Unimpaired	0.10	0.04
	Drug-impaired	0.13	0.05
Mean reaction time for correct-targets, quartile 4	Unimpaired	0.73	0.13
	Drug-impaired	0.84	0.17
Standard deviation of reaction time for correct-targets, quartile 4	Unimpaired	0.16	0.06
	Drug-impaired	0.25	0.19

Significant differences based on impairment were found for the other three tasks as well. For the VPVT, performance differences were found between the two groups, with the drug-impaired group having significantly more missed responses (*M* = 3.92, *SD* = 4.03) than the unimpaired group (*M* = 1.24, *SD* = 2.17), *F*(1,28) = 5.52, *p* < 0.05 (no physiologic differences were revealed). Similar to the performance differences observed for the VPVT, the APVT showed significant differences on missed responses between groups with the drug-impaired group missing more responses (*M* = 8.43, *SD* = 7.94) than the unimpaired group (*M* = 1.32, *SD* = 2.87), *F*(1,34) = 14.82, *p* < 0.001. In addition, during the APVT, the drug-impaired group had significantly more lapses (failure to respond for 3 s or longer, *M* = 5.5, *SD* = 5.17) than the unimpaired group (*M* = 0.5, *SD* = 1.19), *F*(1,34) = 19.29, *p* < 0.001. Neurophysiological differences for the APVT revealed that overall Central Theta (C3, Cz, C4; 3–7 Hz) was higher in the drug-impaired group (*M* = 3.43, *SD* = 0.46) in comparison to the unimpaired group (*M* = 3.19, *SD* = 0.23), *F*(1,34) = 4.25, *p* < 0.05. During the SIR task, the drug-impaired group also showed greater variability in reaction times (*M* = 0.18, *SD* = 0.05) compared to the unimpaired group (*M* = 0.13, *SD* = 0.05), *F*(1,33) = 11.23, *p* < 0.01, but no significant differences in accuracy or neurophysiology were found.

### Forward Step Wise Regression

**FIGURE 3:**
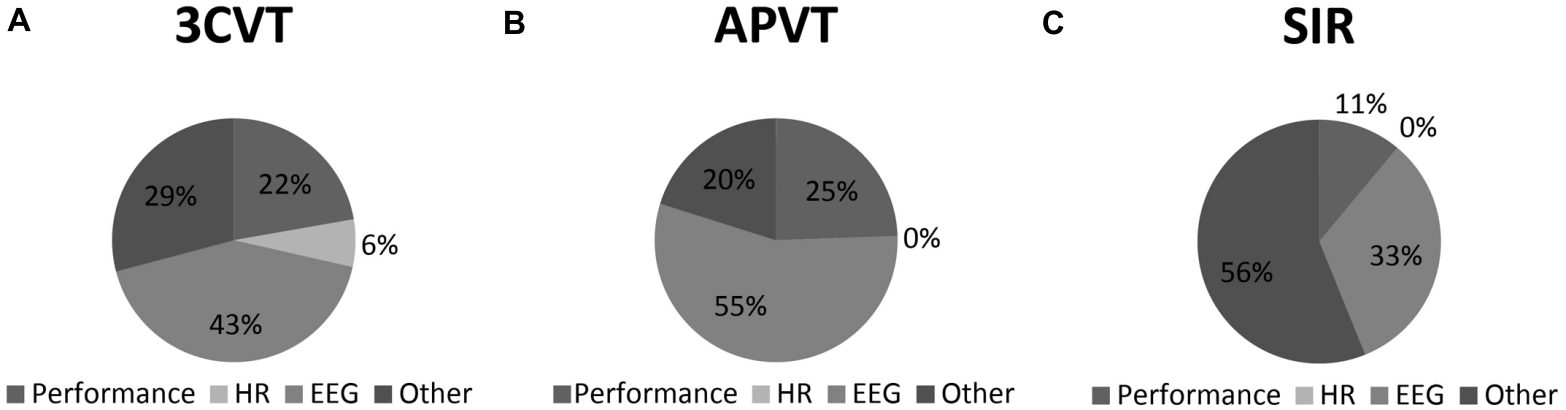
**Standard Deviation of Lateral Position (SDLP) variance explained by HR, EEG, and Performance metrics within the (A) 3CVT; (B) APVT; and (C) SIR task**.

As individual M-AMP neurophysiologic and performance metrics were not substantially informative in identifying impairment, we explored whether the combination of multiple metrics could best predict impaired driving performance. To this end, we applied a forward step-wise regression of the M-AMP metrics within each M-AMP task onto SDLP and LnDP. For SDLP, 3CVT metrics explained 70.9% of variance, *F*(14,18) = 7.62, *p* < 0.05, primarily by hemispheric differences in parietal Alpha (8–13 Hz) (17.5%) and frontal (F4) Gamma (25–40 Hz, 8.0%). Overall, 70.9% of variance was explained by: EEG metrics (42.3%), elevated HR (6.3%), and performance (22.2%). In contrast, the VPVT metrics were not statistically significant in explaining SDLP variability. However, the APVT metrics explained 79.9%, with lapses explaining the most variability at 17.5%, and with EEG metrics making up the remainder (55.4%), *F*(15,19) = 11.24, *p* < 0.01. Finally, SIR metrics explained only 43.9% of SDLP variance, primarily with hemispheric differences (32.8%) at the frontal (F3 and F4) and central (C3 and C4) sites within Slow Alpha (8–10 Hz) and Beta (13–30 Hz). Performance metrics made up the remainder (11.1%), *F*(4,29) = 4.92, *p* < 0.05. These data are presented in **Table 4** and visualized in **Figure 3**.

**Table 4:** Standard Deviation of Lateral Position (SDLP) regression models.

	Standard deviation in lane departures (SDLP)		
	Metric	*F*	Adj. *R*^2^
15*90**3CVT**	Parietal hemispheric difference, Alpha (8–13 Hz)	6.19ˆ*	0.18
	F4 Gamma (25–40 Hz)	4.06ˆ*	0.08
	Standard deviation of reaction times to non-targets	4.6ˆ*	0.07
	Heart rate (HR)	4.53ˆ*	0.06
	Frontal hemispheric difference, Delta (1–3 Hz)	5.91ˆ*	0.07
	POz Slow Theta (3–5 Hz)	6.04ˆ*	0.06
	Reaction time to non-targets, quartile 4	6.09ˆ*	0.05
	Incorrect non-target rate, quartile 1	3.3	0.02
	Incorrect non-target rate, quartile 4	8.26ˆ**	0.04
	Accuracy overall, quartile 4	3.84ˆ*	0.02
	Overall Gamma (25–40 Hz)	5.77ˆ*	0.02
	Overall hemispheric differences, Beta (13–30 Hz)	7.5ˆ*	0.02
	Incorrect interference rate, quartile 4	5.57ˆ*	0.01
	Accuracy non-target Rate, quartile 1	7.62ˆ*	0.01

15*90**APVT**	Lapses, 6 s or longer	7.03ˆ*	0.18
	Central hemispheric differences, Gamma (25–40 Hz)	3.3ˆ*	0.07
	Central hemispheric differences, Delta (1–3 Hz)	3.07ˆ*	0.06
	Frontal hemispheric difference, Fast Alpha (10–13 Hz)	2.75	0.05
	Lapses, 3 s or longer	4.5ˆ*	0.07
	C4 Theta (3–7 Hz)	3.74	0.05
	Cz Fast Alpha (10–13 Hz)	6.95ˆ*	0.08
	Fz Delta (1–3 Hz)	4.2	0.04
	C4 Sigma (12–15 Hz)	3.4	0.03
	POz Gamma (25–40)	3.39	0.03
	Central hemispheric differences, Alpha (8–13 Hz)	4.37ˆ*	0.03
	Overall hemispheric differences, Delta (1–3 Hz)	3.91ˆ*	0.02
	Parietal hemispheric difference, Sigma (12–15 Hz)	4.73ˆ*	0.03
	C4 Delta (1–3 Hz)	7.15ˆ*	0.03
	F3 Gamma (25–40 Hz)	11.24ˆ**	0.03

4*90**SIR**	Frontal hemispheric difference, Slow Alpha (8–10 Hz)	7.42ˆ*	0.19
	Incorrect target rate	4.94ˆ*	0.11
	Frontal hemispheric difference, Beta (13–30 Hz)	2.37	0.05
	Central hemispheric differences, Beta (13–30 Hz)	4.92ˆ*	0.09

*^∗^p < 0.05; ^∗∗^p < 0.01; Adj. R^2^ = adjusted R^2^ value of the model when the variable was entered into the model.*

**FIGURE 4:**
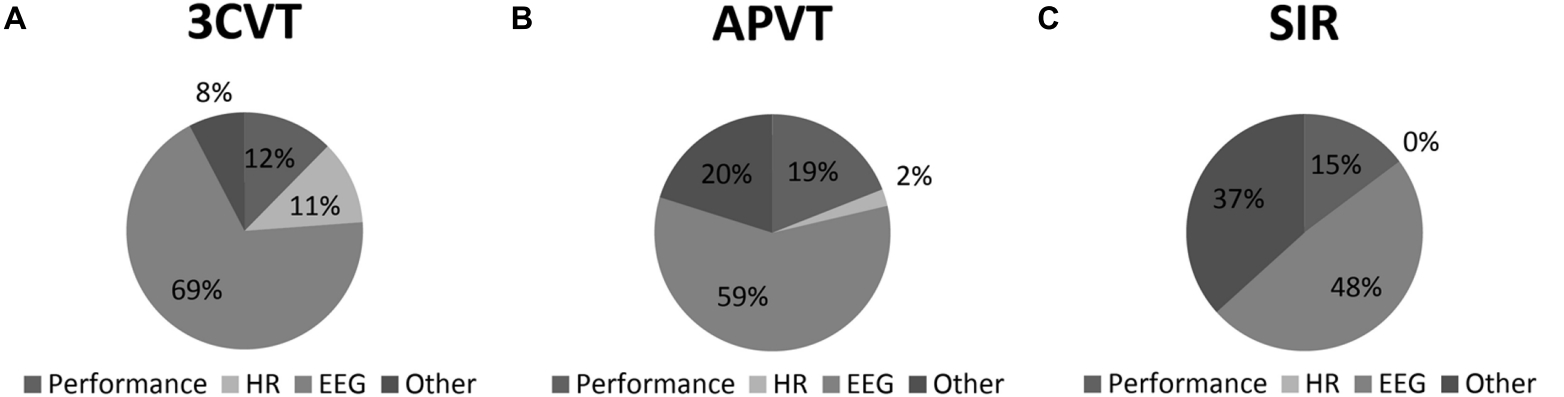
**LnDP variance explained by HR, EEG, and Performance metrics within the (A) 3CVT; (B) APVT; and (C) SIR task**.

For the LnDPs, the 3CVT metrics were the most explanatory, at 92.3%, *F*(13,19) = 10.66, *p* < 0.01, that relied on hemispheric parietal (P3 and P4) differences in Gamma (25–40 Hz) (19.5%) and HRV during the third quartile of the 3CVT (11.3%), with other EEG metrics making up the majority (68.5%) of the variance explained, and performance contributing 12.4%. Once again, the VPVT had a statistically insignificant model and thus was not valid in explaining variability in LnDPs. The APVT metrics, again, had high explanatory value *F*(14,19) = 5.18, *p* < 0.05, with EEG making up the majority of the metrics (58.5%) at various locations and bandwidths. Similar to SDLP, performance metrics within the APVT had a significant explanatory role (19.0%), relying primarily on lapses. HRV metrics within the APVT, though small (2.31%), also contributed to explaining lane departures, such that decreases in HRV were associated with increases in the variance in lane departures. Finally, the SIR metrics explained 63.3%; *F*(5,27) = 6.7, *p* < 0.05; relying on hemispheric differences (48.5%) across Alpha, Sigma, Beta, and Gamma bandwidths, as well as performance (14.8%). These data are presented in **Table 5** and visualized in **Figure 4**.

**Table 5:** LnDP regression models.

	Lane departures (LnDPs)		
	Metric	*F*	Adj. *R*^2^
15*90**3CVT**	Parietal hemispheric difference, Gamma (25–40 Hz)	6.81ˆ*	0.2
	HRV, quartile 3	4.46ˆ*	0.11
	F4 Delta (1–3 Hz)	4.24ˆ*	0.1
	F4 Slow Theta (3–5 Hz)	13.52ˆ**	0.21
	Standard deviation of reaction times to Targets, quartile 3	5.72ˆ*	0.07
	Frontal hemispheric difference, Fast Theta (5–7 Hz)	10.31ˆ**	0.08
	Midline (Fz, Cz, POz) Delta (1–3 Hz)	10.21ˆ**	0.05
	P3 Fast ThetaF (5–7 Hz)	6.17ˆ*	0.03
	Reaction time to non-targets, quartile 4	3.31	0.01
	Reaction time to non-targets, quartile 2	7.52ˆ*	0.02
	Parietal hemispheric difference, Alpha (8–13 Hz)	10.1ˆ**	0.02
	Frontal (Fz, F3, F4) Fast Theta (5–7 Hz)	7.87ˆ*	0.01
	Reaction time to interference stimuli, quartile 3	7.73ˆ*	0.01

15*90**APVT**	Lapses, 6 s or longer	7.53ˆ**	0.19
	F3 Gamma (25–40 Hz)	7.54ˆ**	0.16
	Central hemispheric differences, Sigma (12–15 Hz)	7.45ˆ*	0.13
	Frontal hemispheric difference, Fast Alpha (10–13 Hz)	2.51	0.04
	F3 Delta (1–3 Hz)	4.38ˆ*	0.06
	Cz Fast Theta (5–7 Hz)	2.9	0.03
	C4 Delta (1–3 Hz)	3.18	0.03
	Central hemispheric differences, Alpha (8–13 Hz)	5.91ˆ*	0.05
	Central hemispheric differences, Beta (13–30 Hz)	5.1ˆ*	0.04
	P3 Gamma (25–40 Hz)	3.1	0.02
	Parietal hemispheric difference, Sigma (12–15 Hz)	2.25	0.01
	Parietal (Pz, P3, P4) Fast Alpha (10–13 Hz)	3.15	0.02
	C4 Gamma (25–40 Hz)	2.63	0.01
	HRV	5.18ˆ*	0.02

5*90**SIR**	Frontal hemispheric difference, Slow Alpha (8–10 Hz)	6.95ˆ*	0.18
	Incorrect target rate	6.66ˆ*	0.15
	Overall hemispheric differences, Beta (13–30 Hz)	5.69ˆ*	0.11
	POz Gamma (25–40 Hz)	6.41ˆ*	0.1
	Overall hemispheric differences, Sigma (12–15 Hz)	6.7ˆ*	0.09

*^∗^p < 0.05; ^∗∗^p < 0.01; Adj. R^2^ = adjusted R^2^ value of the model when the variable was entered into the model.*

### Discriminant Function Analysis (DFA)

In order to further determine if the variables found in the regression analyses could identify impairment risk, we entered these variables into a 2-class (drug-impaired/unimpaired) linear discriminate function analyses. While highly preliminary, the results are promising from the leave one out cross validation (LOOCV) method. The function was identified using the LOOCV method to examine the generalizability of the model. LOOCV uses one observation from the N = 30–32 as the validation data in order to use it as testing data for the validation model, while the rest of the data is used as training data. This process is then repeated for each observation in the dataset so that each sample is used once as the validation data. This, in turn, creates the function prediction from the regression variables. By using the significant variables from the 3CVT, APVT, and SIR regressions on SDLP, this analysis resulted in models that were 63.1, 87.0, and 76.8% accurate, respectively. For the 3CVT and SIR, only the first four variables from the regression were used because it was found that adding more variables actually reduced accuracy of the model. However, for the APVT, 13 variables were needed to obtain the aforementioned level of accuracy. Using variables identified in the Lane departure regressions, we also found promising predictive results of impairment. Again, the VPVT could not predict impairment due to an insignificant regression model. However, the 3CVT, APVT, and SIR offered promising predictive models that were 72.7, 73.4, and 77.0% accurate, respectively, with 9, 17, and 6 variables.

### Machine Learning Approach – Boosting

Recognizing that the series of regressions that provided the variables to be used within the DFA has the propensity of introducing a biased estimate of the population variance accounted for, it is likely that the aforementioned models could overfit the predictor variables due to the small sample size. To account for this, an adaptive boosting approach, AdaBoost (*n* = 20 boosting rounds) was also applied to the dataset in a leave-one-out cross-validation manner. This approach aims to identify the strong boosted classifier that predicts impairment risk (drug-impaired/unimpaired). Because the VPVT did not have statistically significant variables from the regressions, this task was not incorporated into the boosting analysis. However, the leave-one-out cross-validation of the remaining three tasks’ metrics were able to classify “impaired” vs. “unimpaired” based on lane maintenance. Respectively, the 3CVT, APVT, and SIR were found to have 58.06, 80.56, and 71.43% accuracy in distinguishing such classifications.

## Discussion

Driving under the influence of drugs, especially benzodiazepines, is not only hazardous for the driver, but everyone on the road (Walsh et al., 2004; NHTSA, 2010; Dassanayake et al., 2011; Li et al., 2013; Brady and Li, 2014). DREs are least likely to accurately recognize driving impairments due to nonalcoholic, CNS depressants (including benzodiazepines), as compared to impairment associated with other classes of drugs (Smith et al., 2002). Validation of the existing criteria for determining impaired driving, as well as the addition of new criteria, are needed to assist DREs in conducting such assessments. Studies have shown promise for utilizing cognitive, behavioral, and neurophysiological signatures associated with benzodiazepine use and impairment to aid in such investigations (Hendler et al., 1980; O’Hanlon et al., 1982; Fernández-Guardiola et al., 1984; Buchsbaum et al., 1985; Smiley and Moskowitz, 1986; Linnoila et al., 1990; Bond et al., 1992; Van Laar et al., 1992; Kuitunen, 1994; Kožena et al., 1995; Suzuki et al., 1995; Münte et al., 1996; Rush and Griffiths, 1997; Bocca et al., 1999; Vanakoski et al., 2000; Riba et al., 2001; Van Laar et al., 2001; Verster et al., 2002; Barker et al., 2004; Eoh et al., 2005; Snyder et al., 2005; Staner et al., 2005; Leufkens et al., 2007). Research has even shown laboratory tasks to be directly related to SDLP, a widely used measure of driving performance (Verster et al., 2002). However, these tasks alone cannot predict much of the variability in driving performance (Verster and Roth, 2012). It is possible that the addition of neurophysiology can help explain more of this variance. Therefore, the current study sought to determine whether all three classes of correlates could be leveraged as a new approach for validating and suggesting improvements to the current DRE evaluations.

The current findings, in agreement with previous work, indicate that SDLP and LnDP are good indicators of impairment and that the observation of poor lane position maintenance provides justification for conducting a DRE evaluation (O’Hanlon et al., 1982; NHTSA, 1997; Bocca et al., 1999; Van Laar et al., 2001; Verster et al., 2002; Leufkens et al., 2007). Our analyses were able to pinpoint specific driving conditions that make lane position maintenance especially difficult for those under the influence of benzodiazepines. These conditions include open stretches of the interstate or highway, as well as rural roadways. Thus, lane position difficulties under these driving conditions may serve as particularly useful criteria for officers to detect driving impairments associated with benzodiazepines.

Though the results presented herein are in agreeance with past literature, as previously mentioned, this dynamic environment did introduce the likelihood for higher SDLP values. This is important to note because three of the five statistically significant segments revealed from the ANOVAs were designed to introduce more challenging maneuvers than observed in previously published work. This is particularly true for the interstate highway event. Although straight, this segment of the driving course contains slower moving traffic, which causes the drivers to circumnavigate the slower traffic by performing two lane changes. These lane changes produce higher levels of SDLP relative to the other events within the simulator. Similarly, the rural events that yielded significant differences in the ANOVA’s also have elevated SDLP values. This is experienced in the Rural-Transition to Dark segment due to the curvy nature of the roadway and the short duration of time from dusk to dark while on this roadway. The Rural-Straight portion of the drive also introduces variances in SDLP values that are unlike those of past studies. Even though this segment is straight, SDLP is elevated due to the changes in participant’s alertness/wakefulness as this is one of the last segments of the drive. Prior work has yielded similar results; Brown et al. (2014) used this same scenario and showed that even during daytime drives, sleepiness increased, as measured by the Stanford Sleepiness Scale, over the course of the drive and SDLP was elevated relative to earlier in the drive. These three events have been specifically included in this evaluation due to the fact that they represent more challenging environments. Therefore, they are more likely to highlight, and be sensitive to, drug effects despite not conforming to the typical environments in which they have been historically reported. On the other hand, the other two statistically significant segments revealed within the SDLP ANOVAs have values similar to previously suggested guidelines (Brookhuis et al., 2003). While the urban general section does contain a portion where the roadway is slightly curved, the urban green light segment is similar to more commonly used driving simulator environments and may best serve as a direct comparison to past literature. The fact that the urban green light segment is both environmentally similar to past literature and yields similar SDLP values suggests that the performance of participants in both conditions are comparable to that of those reported in previous studies.

The current study expanded on the findings that benzodiazepines impair lane maintenance behaviors by exploring the use of a neurocognitive assessment for explaining the lane maintenance errors of drivers under the influence of benzodiazepines. ANOVA results revealed behavioral findings consistent with this field of literature, such as increased reaction times during the 3CVT (Fernández-Guardiola et al., 1984; Suzuki et al., 1995; Münte et al., 1996; Verster et al., 2002; Barker et al., 2004; Snyder et al., 2005; Leufkens et al., 2007) and impaired vigilance on the APVT and VPVT as indicated by the missed responses and lapses (Kožena et al., 1995). However, we did not find any indications of memory impairment during the SIR. The EEG ANOVA results indicated a reduction in parietal Gamma (25–40 Hz), an elevation in Midline Alpha (8–10 Hz) during the 3CVT, and Central Theta elevation during the APVT. The central elevation in theta is consistent with findings that increases in both Delta (1–3 Hz) and Theta (3–7 Hz) are positively correlated with fatigue while driving and that benzodiazepines are associated with increased fatigue (Lal and Craig, 2002; Campagne et al., 2004). In contrast, the elevated Midline Alpha is inconsistent with most studies that find benzodiazepines decrease Alpha (8–13 Hz) in the frontal, temporal, and occipital regions (Buchsbaum et al., 1985; Bond et al., 1992; Staner et al., 2005). However, suppression of Alpha can also be an adaptive response to increasing simulation difficulty, indicating that a failure to suppress Alpha may be a sign of failure to adapt by those under the influence of a benzodiazepine (Eoh et al., 2005).

The regression and discriminate function analyses are more consistent with prior work indicating that Beta (Hendler et al., 1980; Buchsbaum et al., 1985; Bond et al., 1992; Staner et al., 2005), Frontal Delta (Buchsbaum et al., 1985; Staner et al., 2005), and Alpha over multiple regions (Buchsbaum et al., 1985; Bond et al., 1992; Staner et al., 2005) are associated with benzodiazepine use. These findings also support the combination of neurophysiology and performance in objectively assessing and predicting impairment due to benzodiazepines, as past studies failed to explain variability in SDLP using performance metrics alone (Verster and Roth, 2012). To this end, the current data indicate that combining performance metrics with neurophysiology can explain up to 79.9% of the variance in SDLP using APVT metrics alone. However, the combination of metrics within the regression analyses indicated that HR measures were only significant in explaining SDLP impairment within the 3CVT, and not in any of the other tasks. Herein, results were in agreeance with past literature, as HR during this task did increase as a result of the benzodiazepine (Muzet et al., 1981; DiMicco, 1987; Ueda et al., 2013) and was a significant predictor of SDLP. This approach yielded promising results for lane departures as well. In comparison to the SDLP results, we were able to explain more of the variance in lane departures with 3CVT metrics, explaining 92% of the variance in this measure. Similar to the results seen for SDLP, HRV was shown to have a significant explanatory power within the 3CVT task for assessment of lane departures. HRV measures showed significant explanatory power within the APVT task as well, which supports the results from previous studies showing that benzodiazepines decrease HRV (Adinoff et al., 1992; Agelink et al., 2002), decreases in HRV are related to increases in drowsiness related driving errors (Michail et al., 2008), and that decreased HRV is associated with cognitive and behavioral impairments (Hansen et al., 2003). The discriminate function classification and boosting results were even more promising, respectively, showing up to 87 and 80% accuracy in identifying impairment likelihood based on neurophysiology and performance within cognitive tasks. While preliminary, and in need of replication in larger confirmatory studies, these results indicate the promise of this approach as a way for researchers to gain further insight into the correlates of drug impaired driving and consequently aid in the optimization of assessments of benzodiazepine impaired driving.

While promising, this study does have limitations that should be taken into account when interpreting our results. The simulated environment utilized herein contains more diverse driving scenarios than that of previous studies in order to more closely resemble real-world driving. While results from this design may have greater external validity, they do not easily lend to comparisons with past findings. Future studies utilizing the current approach could employ both types of driving environments in order to enable such comparisons. In regards to demographics, the majority of participants were young males, just as most drugged drivers are also young males (Lacey et al., 2009). This approach requires validation across genders and age groups to determine whether the models will generalize across a larger population. Additionally, we utilized a within subject design (to reduce individual differences across conditions), but with the small sample size, this may lead to an even greater likelihood of overfitting the regression and discriminant function analyses. Future research is needed to verify whether these findings can be replicated with a larger sample size. Replication and expansion of our design is also necessary in terms of dosing levels. Since participants were only dosed with 1 mg of alprazolam in the current study, our findings may only represent deficits associated with moderate to low doses of this benzodiazepine. A dose-response study should be conducted to verify whether these same correlates can predict, and classify, driving impairment due to a range of dosing levels and to reveal whether other correlates may better explain variances in performance due to a wide range of doses. Other benzodiazepines, as well as an array of CNS depressants outside of the benzodiazepine class, should also be examined to determine if the effects found within this study are limited to alprazolam, or are true for all benzodiazepines and CNS depressants. Furthermore, conditions that are known to interact with CNS depressant use, such as sleep deprivation and alcohol use, should be examined to see how their interactions affect driving impairment. Future studies should expand on these findings to include broader demographic diversity, greater sample size, dose response, administration of different benzodiazepines, and the use of other types of CNS depressants. Taking these suggested limitations and improvements into consideration, future studies should target a means of taking the research beyond the scope of a simulated environment in order to better provide the much needed support for law enforcement in ensuring public health and safety for all drivers.

## Conflict of Interest Statement

Authors Robin R. Johnson and Chris Berka are share holders in Advanced Brain Monitoring, which may benefit financially from the publication of these data. The other authors declare that the research was conducted in the absence of any commercial or financial relationships that could be construed as a potential conflict of interest.
